# Blood neutrophil counts in HIV-infected patients with cryptococcal meningitis: Association with mortality

**DOI:** 10.1371/journal.pone.0209337

**Published:** 2018-12-31

**Authors:** Abdu Kisekka Musubire, David B. Meya, Joshua Rhein, Graeme Meintjes, Paul R. Bohjanen, Edwin Nuwagira, Conrad Muzoora, David R. Boulware, Kathy Huppler Hullsiek, the COAT and ASTRO trial teams

**Affiliations:** 1 Infectious Disease Institute, College of Health Sciences, Makerere University, Kampala, Uganda; 2 Department of Medicine, School of Medicine, College of Health Sciences, Makerere University and Mulago Hospital Complex, Kampala, Uganda; 3 Division of Infectious Diseases & International Medicine, Dept. of Medicine, University of Minnesota, Minnesota, Minneapolis, United States of America; 4 Centre for Infectious Diseases Research in Africa, Institute of Infectious Disease and Molecular Medicine, University Cape Town, Cape Town, South Africa; 5 Department of Medicine, Mbarara University of Science and Technology, Mbarara, Uganda; 6 Division of Biostatistics, School of Public Health, University of Minnesota, Minnesota, Minneapolis, United States of America; Oklahoma State University, UNITED STATES

## Abstract

**Background:**

The mortality from cryptococcal meningitis remains high, despite the availability of antiretroviral therapy (ART) and amphotericin-based fungal regimens. The role of neutrophils in cryptococcosis is controversial. Our objective was to examine the association between blood neutrophil counts and outcomes in terms of mortality, the incidence of bacterial infections (including Mycobacterium tuberculosis) and hospitalization among HIV-infected patients presenting with cryptococcal meningitis.

**Methods:**

We used data from participants from the Cryptococcal Optimal ART Timing (COAT) trial (2010–2012; Uganda and South Africa) and the Adjunctive Sertraline for Treatment of Cryptococcal Meningitis (ASTRO-CM) trial (2013–2017; Uganda). We estimated 30-day mortality risk with Cox proportional hazards models by baseline neutrophil counts (a) on a continuous scale and (b) with indicators for both relatively high (> 3,500 cells/mm^3^) and low (≤ 1,000 cells/mm^3^) counts. Follow-up neutrophil counts from the COAT trial were used to examine the time-dependent association of neutrophil counts with 12-month mortality and rehospitalization.

**Results:**

801 participants had an absolute neutrophil value at meningitis diagnosis. The median baseline absolute neutrophil count was 2100 cells/mm^3^ (IQR, 1400 to 3300 cells/mm^3^). Baseline neutrophil count was positively associated with 30-day mortality (adjusted hazard ratio = 1.09, 95%CI, 1.04–1.13, per 1000 cells/mm^3^ increase; p<0.001). Baseline absolute neutrophil counts ≤ 1000 cells/mm^3^ did not have increased risk of 30-day mortality compared to those with baseline neutrophils of 1001–3500 cells/mm^3^; however, baseline >3500 cells/mm^3^ had significantly increased risk, with an adjusted hazard ratio of 1.85(95%CI, 1.40–2.44; p<0.001). Among the COAT participants with follow-up neutrophil data, there was a strong association between time-updated neutrophil count and 12-month mortality (adjusted hazard ratio = 1.16, 95% CI 1.09–1.24; p<0.001.

**Conclusion:**

Higher blood neutrophil counts in HIV-infected patients with cryptococcal meningitis were associated with mortality. Neutrophils role requires further investigation as to whether this may be a mediator directly contributing to mortality or merely a marker of underlying pathologies that increase mortality risk.

## Introduction

The mortality from cryptococcal meningitis remains high at 6 months, about 50% in several studies despite the availability of antiretroviral therapy (ART) and improved fungal regimens. Most of the mortality occurs early during the antifungal initiation and consolidation phase.[1, 2] Cryptococcal infection occurs by inhalation through the respiratory system where the fungal organisms multiply in the lungs, leading to infection.[3] *Cryptococcus* then escapes from the lung into the bloodstream where the yeast persists before invading the brain.[4]

Neutrophils have been shown to be efficient in killing several strains of *C*. *neoformans* in vitro.[5] Neutrophil recruitment into the lungs is observed during the early phase of cryptococcal infection.[6] *Cryptococcus* has demonstrated chemotactic activity on the neutrophils in vitro,[7] though paradoxically, the capsular polysaccharide glucuronoxylomannan (GXM) has been shown to inhibit neutrophil migration [8] and block neutrophil binding of fungal cells. [9]

The role of neutrophils in cryptococcal infection is still debated as either noncontributory or even detrimental because cryptococcosis is seen primarily in patients with dysfunctions in adaptive immunity and not in patients with neutropenia.[4] Animal studies have shown improved survival of mice transiently depleted of neutrophils at the time of pulmonary infection with *Cryptococcus* [10, 11] which appears to be caused by the absence of neutrophil-mediated inflammation.[12]

However, other mouse models have supported the protective role on neutrophils with an increased number of neutrophils associated with lesser fungal burden.[13, 14] In vivo imaging studies have demonstrated neutrophils directly removing *Cryptococcus* from the brain vasculature.[14, 15] Furthermore, the augmentation of neutrophil defenses by administration of granulocyte colony stimulating factor (G-CSF) to enhance anti-cryptococcal activity has been demonstrated in vitro [16], animal models[17], as well as in persons living with AIDS. [18, 19] A deficiency of myeloperoxidase, an enzyme with the most abundant expression in neutrophils, has been found to significantly shorten the survival of *Cryptococcus* infected mice with higher brain fungal burden. [20]

Neutrophil counts, either high or low, may have prognostic implications in Human immunodeficiency virus infection (HIV) patients with cryptococcal meningitis. Our objective was to examine the association between blood neutrophil counts and outcomes in terms of mortality, the incidence of bacterial infections including Mycobacterium tuberculosis and hospitalization among HIV-infected patients presenting with cryptococcal meningitis.

## Materials and methods

Data from participants who were enrolled in either the Cryptococcal Optimal ART Timing (COAT) trial or the Adjunctive Sertraline for Treatment of Cryptococcal Meningitis (ASTRO-CM) trial (pilot and randomized phases) were used for this analysis. The COAT trial enrolled Ugandan and South African HIV-infected, ART-naive individuals diagnosed with a first episode of cryptococcal meningitis from 2010–2012, as previously described.[1] The ASTRO-CM pilot trial was an open-label dose-finding study of adjunctive sertraline conducted in Uganda from August 2013 to August 2014 followed by a randomized treatment trial from August 2014 through May 2017.[21] Both studies excluded pregnant women and patients <18 years of age. The diagnosis of cryptococcal meningitis was by cerebrospinal fluid (CSF) cryptococcal antigen testing at Mulago National Referral Hospital, Kampala, Uganda (COAT and ASTRO-CM), Mbarara Hospital, Mbarara, Uganda (COAT and ASTRO-CM), or G.F. Jooste Hospital, Cape Town, South Africa (COAT). All participants in both trials received combination induction therapy with amphotericin B deoxycholate (0.7–1.0 mg/kg/day) and fluconazole 800 mg/day. In addition, we administered sertraline (100–400 mg/day) to all participants in the ASTRO-CM pilot trial. ASTRO trial participants were randomized to receive either sertraline (400 mg/day) or placebo during induction therapy. Blood samples were taken in EDTA bottle and analyzed within 12 hours after the venepuncture. A complete blood count was done on a Sysmex XS-1000*i* Automated Haematology Analyzer. However, thin blood films were not done. Neutrophil levels were obtained at the time of cryptococcal meningitis diagnosis in both trials. Follow-up neutrophil data was measured for COAT trial participants at 1, 2, 5, 9, 13, and 27 weeks from cryptococcal diagnosis.

We summarised the absolute neutrophil counts at cryptococcal meningitis diagnosis (all participants) and subsequent weeks (COAT participants only). Three groups were categorized by baseline neutrophil count (≤1000, 1001–3500, and >3500 cells/mm^3^). Neutropenia was defined as absolute neutrophil count ≤1000 cells/mm^3^ in line with the Division of AIDS (DAIDS) Table for Grading the Severity of Adult and Pediatric Adverse Events. The category formed by baseline neutrophil counts > 3500 cells/mm^3^ was based on the upper quartile of the data from all participants. Baseline characteristics and outcomes within 30 days were presented and compared between the three groups with Chi-squared and Kruskal-Wallis tests as appropriate. Outcomes considered included 30-day mortality (including sepsis and TB-related mortality) and the incidence of infection and tuberculosis. The infections were documented if they fulfilled the DAIDs toxicity table grade 3 and above with investigations done at physicians’ discretion. Participants with suspected sepsis according to the systemic inflammatory response syndrome (SIRS) criteria [22] had blood cultures in the COAT study. However, this was not possible in the ASTRO study because of budgetary limitations, and most of the diagnoses were based on clinical criteria. Tuberculosis was investigated by chest radiographs and abdominal ultrasound, sputum microscopy for acid-fast bacilli in the COAT trial and Xpert MTB/RIF (Cepheid, Sunnyvale, CA) in the ASTRO trial.[23, 24] We did not perform mycobacteria blood cultures.

To model the association between baseline neutrophil count and 30-day mortality, cohort-adjusted and fully-adjusted Cox proportional hazards models were examined with (a) neutrophil count on a continuous scale (per 1000 cells/mm^3^), and (b) comparing the three baseline neutrophil groups (comparing the lower and upper groups with the 1001–3500 cells/mm^3^ group). All models were adjusted for study cohort and are summarized with hazard ratios and 95% confidence intervals. The fully adjusted Cox models considered baseline prognostic variables known to be associated with cryptococcal-related deaths: Glasgow coma scale, sex, ART status, CSF opening pressure, and CSF quantitative culture.

Neutrophil counts were obtained during follow-up for the COAT study only. For COAT participants with follow-up blood neutrophil data available, unadjusted and fully adjusted Cox proportional hazards models considering time-dependent neutrophil count (per 1000 cells/mm^3^ increase) were used to examine the relationships between neutrophil count and 12-month mortality and rehospitalization.

All research participants or their surrogate provided written informed consent. Ethical approval occurred from the Uganda National Council of Science and Technology (UNCST), Mulago Hospital Research and Ethics Committee, Makerere University Institutional Review Board, University of Cape Town institutional Review Board and University of Minnesota.

## Results

### Baseline characteristics

The baseline characteristics of participants are described in Table 1, overall and by baseline blood neutrophil category (≤1000, 1001–3500, or > 3500 cells/mm^3^). Baseline neutrophil data was available for 801 participants: 238 (30%) who were eligible for COAT, and 563 (70%) who were eligible for ASTRO. The median (IQR) blood neutrophil count was 2100 (1400, 3300) cells/mm^3^. The median (IQR) age was 35 (30–40), with male sex accounting for 61% of the participants. The proportion with a Glasgow coma scale (GCS) <15 (altered mental status) was 22%, 41% and 50% for the baseline neutrophil groups ≤ 1000, 1001–3500 and > 3500 cells/mm^3^, respectively (p<0.001). The median (IQR) CD4 count was 18 (7, 54) cells/mm^3^, and was significantly lower in the baseline neutrophil group ≤ 1000 cells/mm^3^ group (p = 0.02). At cryptococcal meningitis diagnosis, 34% of the participants were on antiretroviral therapy (all from the ASTRO study), and of those 16% were on a zidovudine (AZT)-containing regimen.

The median (IQR) CSF opening pressures were lower in the baseline neutrophil ≤ 1000 group (p = 0.02). Approximately 14% of participants were screened for study enrolment while already on amphotericin, with a median (IQR) of 2 (2–3) doses prior to study screening. C-reactive protein (CRP) was measured in the day 6–11 window for 162 (20%) of participants. For those with a CRP measurement, the medians were 42, 63 and 84 for the baseline neutrophil groups ≤ 1000, 1001–3500 and > 3500 cells/mm^3^, respectively (p = 0.02).

**Table 1:** Baseline demographics by neutrophil group.

	Neutrophils at Screening (cells/mm^3^)				
	Overall	≤ 1000	1001–3500	> 3500	P-value^1^
No. People	801	98	520	183	
Neutrophil count, median cells/mm^3^	2100 [1400, 3300]	750 [590, 910]	1960 [1490, 2510]	4870 [4030, 6060]	
Age, median (IQR) years	35 [30, 40]	35 [30, 38]	35 [30, 42]	35 [29, 41]	0.37
Male gender, N (%)	486 (60.7%)	57 (58.2%)	318 (61.2%)	111 (60.7%)	0.86
Glasgow Coma Scale (GCS) < 15, N (%)	326 (40.8%)	22 (22.4%)	213 (41.0%)	91 (49.7%)	<0.001
TB prevalent at meningitis diagnosis	58 (7.2%)	9 (9.2%)	37 (7.1%)	12 (6.6%)	0.71
Cohort					0.26
COAT	238 (29.7%)	23 (23.5%)	155 (29.8%)	60 (32.8%)	
ASTRO	563 (70.3%)	75 (76.5%)	365 (70.2%)	123 (67.2%)	
HIV Metrics					
CD4 cells/mm^3^	18 [7, 54]	11 [5, 37]	18 [7, 57]	20 [8, 51]	0.02
On antiretroviral therapy	275 (34.3%)	41 (41.8%)	174 (33.5%)	60 (32.8%)	0.24
On zidovudine (AZT) ^2^	43 (15.6%)	6 (14.6%)	31 (17.8%)	6 (10.0%)	0.35
CSF Metrics					
Opening pressure (OP), mmH_2_O	268 [180, 400]	220 [170, 345]	270 [180, 390]	280 [180, 480]	< .01
OP > 250 mmH_2_O	377 (53.9%)	34 (39.5%)	244 (54.2%)	99 (60.7%)	< .01
Quantitative culture^3^, log_10_Colony Forming Unit(CFU)/ml	4.9 [3.8, 5.6]	4.9 [3.7, 5.3]	4.8 [3.7, 5.6]	4.9 [4.2, 5.7]	0.32
Sterile culture	64 (8.3%)	7 (7.4%)	41 (8.2%)	16 (9.1%)	0.88
White blood cells < 5, cells/mm^3^	434 (57.5%)	55 (60.4%)	275 (56.4%)	104 (59.1%)	0.68
On Amphotericin at screening	110 (13.7%)	14 (14.3%)	66 (12.7%)	30 (16.4%)	0.45
Doses of amphotericin^4^	2 [2, 3]	2 [2, 3]	2 [2, 3]	2 [2, 3]	0.45
No. with CRP measured^5^	162	23	100	39	
CRP, mg/L	63.3 [42.3, 121.2]	42.4 [27.2, 79.1]	63.3 [44.6, 119.9]	84.0 [47.1, 140.2]	0.02

^1^Data are median with (P25, P75) or N (%). P-values from Kruskal Wallis or chi-square tests.

^2^Among those on ART.

^3^Not including sterile cultures.

^4^Among those on amphotericin at screening.

^5^Measured 6–11 days after cryptococcal meningitis diagnosis.

### Associations between baseline neutrophils and 30-day outcomes

For those with baseline neutrophils ≤1000, 1001–3500, and >3500 cells/mm^3^, respectively, 30-day mortality was 31%, 31%, and 51% (p<0.001; Table 2). The proportions of those deaths related to sepsis and TB were not significantly different between the baseline neutrophil groups. The incidence (within 30 days) of infections and TB also did not differ between the groups.

**Table 2:** Thirty-day outcomes^1^ by neutrophil group.

	Neutrophils at Screening (cells/mm^3^)			
	≤ 1000	1001–3500	> 3500	P-value^2^
**No. People**	98	520	183	
30-Day Outcome				
Death	30 (30.6%)	159 (30.6%)	94 (51.4%)	<0.001
Related to sepsis	3 (3.1%)	20 (3.8%)	11 (6.0%)	0.38
Related to TB	1 (1.0%)	5 (1.0%)	4 (2.2%)	0.43
Incident infection	5 (5.1%)	18 (3.5%)	5 (2.7%)	0.59
Incident TB	1 (1.0%)	14 (2.7%)	1 (0.5%)	0.16

^1^Data are N (%) with the specified event within 30 days.

^2^P-values are from chi-square tests.

**Fig 1:**
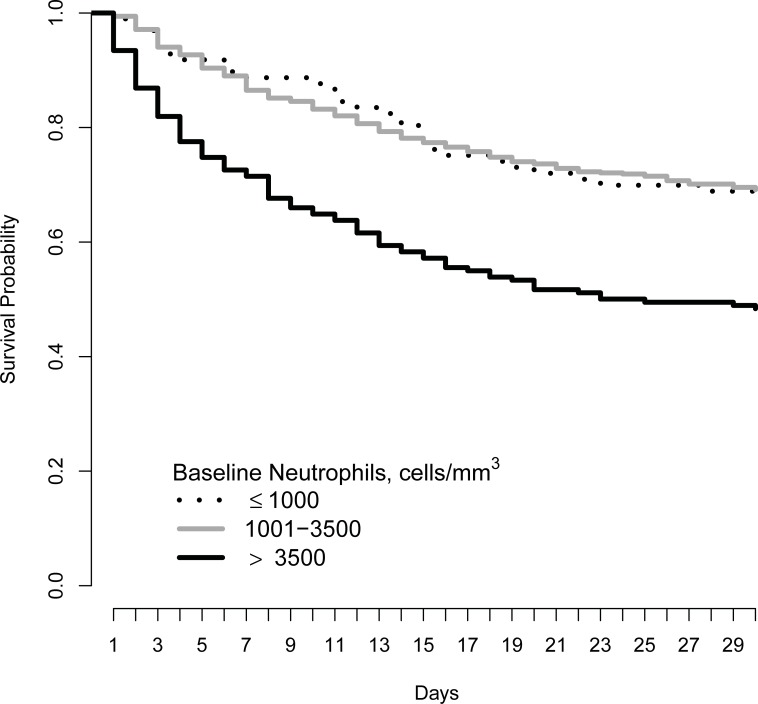
Kaplan-Meier curve describing the survival patients with cryptococcal meningitis by the absolute neutrophil count. The lines represent the absolute neutrophil levels < = 1000 = black dotted, 1001–3500 = grey solid, > 3500 = black solid.

Fig 1 presents a Kaplan-Meier curve for mortality within 30 days for the three-baseline neutrophil groups and shows that the survival probability is lowest for those with baseline neutrophils > 3500 cells/mm^3^. Table 3 presents the results from model associations between neutrophil counts and outcomes. The cohort-adjusted hazard ratio per 1000 cells/mm^3^ increase in baseline blood absolute neutrophil count was 1.10 (95% CI: 1.06, 1.14; p<0.001). Results were similar in a fully adjusted model. Compared to those with baseline blood neutrophils 1001–3500 cells/mm^3^, there was a significantly increased risk of 30-day mortality in those with baseline blood neutrophil count >3500 cells/mm^3^ (cohort adjusted hazard ratio = 2.06, 95%CI, 1.60 to 2.66; p<0.001), but no difference in risk for those with baseline blood neutrophil count ≤1000 cells/mm^3^ (hazard ratio = 1.00, 95%CI, 0.68 to 1.48; p>0.99), with results again being similar with fully adjusted models.

**Table 3:** Model associations between neutrophil counts and outcomes.

		Cohort Adjusted Model^1^		Multivariable Model^2^	
Outcome	Neutrophil Metric	Hazard Ratio (95% CI)	P-value	Hazard Ratio (95% CI)	P-value
30-day mortality	Per 1000 count/mm^3^ increase in baseline neutrophils	1.10 (1.06, 1.14)	<0.001	1.09 (1.04, 1.13)	<0.001
30-day mortality	Compared to baseline neutrophil count of 1001–3500 cells/mm^3^				
	≤ 1000 cells/mm^3^	1.00 (0.68, 1.48)	>0.99	1.01 (0.65, 1.57)	0.96
	> 3500 cells/mm^3^	2.06 (1.60, 2.66)	<0.001	1.85 (1.40, 2.44)	<0.001
12-month mortality^3^	Per 1000 count/mm^3^ increase in time-updated neutrophil counts	1.16 (1.07, 1.24)	<0.001	1.16 (1.09, 1.24)	<0.001
Re-hospitalization^3^	Per 1000 count/mm^3^ increase in time-updated neutrophil counts	0.98 (0.82, 1.17)	0.83	0.87 (0.69, 1.09)	0.23

^1^30-day mortality outcomes are adjusted for the cohort (COAT or ASTRO). Outcomes associated with time-updated neutrophil counts are for COAT only and are unadjusted.

^2^Additionally adjusted for sex, Glasgow coma scale < 15, baseline ART status, baseline opening pressure > 250 mm H_2_O, and baseline quantitative culture.

^3^COAT participants only.

**Fig 2:**
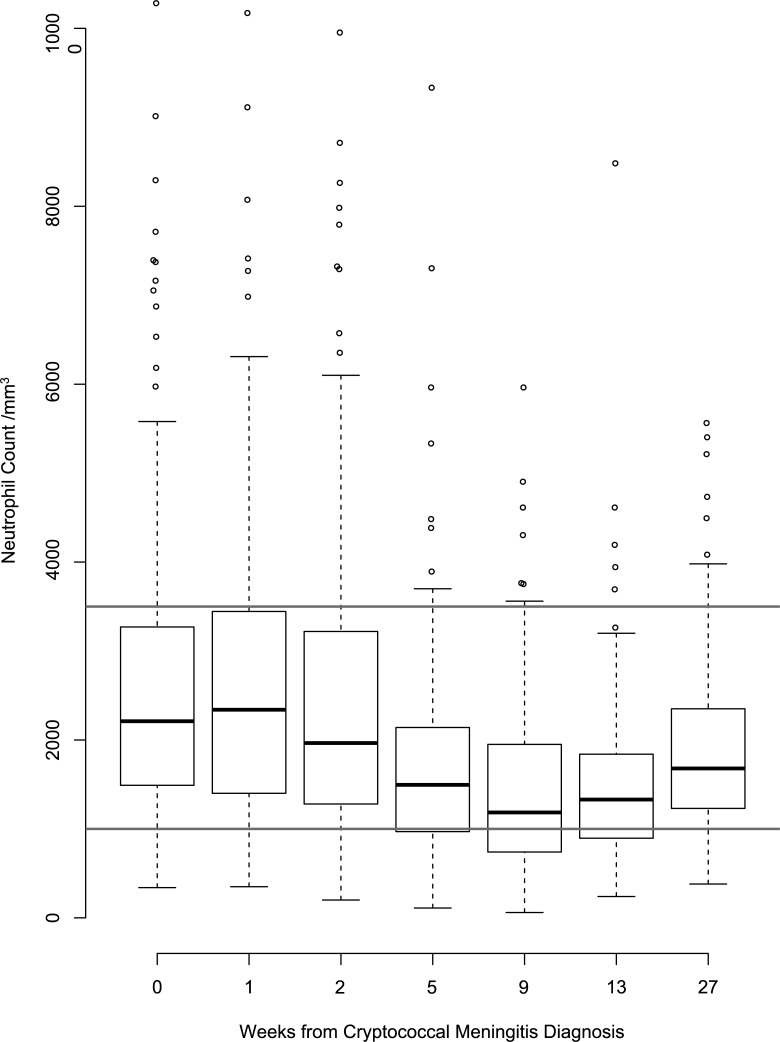
This represents the data from the COAT study reflecting the changing neutrophil count over time. The red horizontal lines represent neutrophil count cut points of 1000 cells/mm^3^ and 3500 cells/mm^3^ (as used in other tables), and the blue points and line represent the means over time.

For the COAT participants with follow-up neutrophil counts measured, Fig 2, presents the neutrophil distribution at screening and weeks 1, 2, 5, 9, 13 and 27. By week 5, when the median neutrophil count decreased from screening, generally, all COAT participants were started on an efavirenz-containing antiretroviral therapy regimen, with 82% also on a zidovudine-containing regimen. Neutropenia (≤ 1000 cells/mm3) in the COAT study at screening and weeks 5, 9 and 13 was 11%, 30%, 44%, and 30%, respectively. While neutropenia increased over time for COAT participants, results in Table 3 from models with time-updated neutrophil counts (per 1000 cell/mm^3^ increase) show that increased neutrophil counts are associated with increased mortality (unadjusted hazard ratio = 1.16, 95% CI 1.07–1.24; p< 0.001). There was no significant association with time-updated neutrophils and re-hospitalization.

## Discussion

This study demonstrated that higher blood neutrophil counts at the baseline in HIV-infected patients with cryptococcal meningitis is associated with increased mortality. This association between higher blood neutrophil count and mortality is maintained even during long-term follow up of these patients. The baseline neutropenia and early decline in blood neutrophil counts during the consolidation phase of antifungal treatment was not associated with increased mortality.

The average baseline neutrophil count was 2,100 cells/mm^3^, comparable to other healthy population-based reference values in other studies done on the African continent.[25–27] The median (range) neutrophil count in healthy volunteers in Malawi was 2,100 (700–4500) cells/mm^3^, thought to be lower than the standard reference values due to the common African-derived ‘‘null” variant (rs2814778) of the Duffy antigen receptor for chemokines (DARC) gene.[28] The average neutrophil count in our study is comparable to that found in HIV-infected Nigerian population where the mean neutrophil count was 2,320 ±1,580 neutrophil/mm^3^.[29]

The prevalence of baseline absolute neutropenia, defined in this study as a neutrophil count <1000/mm^3^, was 12%. This is more than the 7% among HIV-infected American women,[30] but comparable to what was found in a multi-continent study, where the frequencies of neutropenia (absolute neutrophil count <1,300/mm^3^ at the initiation of antiretroviral therapy were 14%.[31] Neither baseline neutropenia nor neutropenia at any time was associated with mortality or hospitalization among cryptococcal patients at 30 days or 12 months of observation. Those with neutropenia had a lower proportion of patients with poor prognostic factors, such as the Glasgow coma scale <15 (altered mental status) or high CSF opening pressures.[32–34] Levine et al also found no association between neutropenia and mortality in HIV-infected women in the US followed up for 7.5 years.[30] Neutropenic mice given a pulmonary *C*. *neoformans* infection survived significantly longer than control mice with mean normal neutrophil count.[10] Neutropenia may be protective in cryptococcal infection because it is associated with alteration of the immune response between Type 1 T helper (Th1) to Type 2 T helper (Th2) response especially in the early phase of the infection.[10, 35–38]

On the contrary, there was a 10% increase in the risk of death within 30 days for every 1000 cells/mm^3^increase in baseline neutrophil count. Patients with an increased baseline blood neutrophil count (>3500 cells/mm^3^) were two-fold more likely to die than those with baseline neutrophil counts between 1001 and 3500 cells/mm^3^. This remained so even after adjusting for known poor prognostic factors in cryptococcal meningitis like low Glasgow coma scale, ART status, CSF opening pressure, and CSF quantitative cultures.[1, 33, 34] The pattern was maintained in the longitudinal follow up of patients where the relatively increased neutrophil count was associated with mortality. Leukocytosis, in general, is associated with cryptococcal mortality. Jarvis et al found that the patients with leukocytosis of >10,000/mm^3^ had 8.7-fold higher odds of mortality. In that South African study, patients with neutrophil counts <500/mm^3^ were excluded.[34] Another study from Taiwan among HIV-negative patients with cryptococcal meningitis also found that patients with leukocytosis >10,000/mm^3^ had 4.3- fold higher odds of mortality even though this effect was lost in multivariate analysis.[39] Scriven et al described a similar pattern of mortality among sixty HIV positive patients with cryptococcal meningitis in South Africa with a higher circulating white blood cell counts among those who died compared with the survivors (median, 6500 cells/mm^3^; IQR, 3900–7400 cells/mm^3^ vs 4400 cells/mm^3^; IQR, 2800–5800 cells/mm^3^; P = .020). Most of this leukocyte difference was attributed to higher relative neutrophil counts with percentages of 76.8% (68.8–82.6) in those who died versus 67.5% (54.6–75.8) in those who survived. [40] Bisson et al published similar results in HIV-infected adults initiating ART with CD4 T-cell counts <50 cells/mm^3^ who were followed up for 48 weeks. The median absolute neutrophil count was 1900/mm^3^ in those who died compared to 1600/ mm^3^ those who survived.[41] One possible explanation for higher neutrophil counts being associated with mortality is the possibility of co-infections. Rajasingham et al have previously reported a15% incidence of nosocomial drug-resistant bacteremia in patients in the COAT study.[42] Kerkhoff et al reported that among HIV-infected patients, having pulmonary tuberculosis was associated with an adjusted risk ratio of 2.6 (95%CI, 1.5–4.5) for having an absolute neutrophil count higher than the median value (p = 0.0006). In that cohort, the adjusted risk ratio was 6.8-fold higher for having tuberculosis when having neutrophilia defined as > 7500 neutrophils/mm^3^.[43] Lowe et al have found that a neutrophilia was associated with a three-fold higher risk of mortality in a predominantly HIV-negative population with tuberculosis.[44] We assessed for tuberculosis in most of our study patients using a clinical case definition, chest X-rays and sputum according to the standard of care at the time and found no association. The other possible cause of mortality in HIV infected patients may be immune reconstitution inflammatory syndrome (IRIS). Elevated CRP levels, which are associated with paradoxical IRIS[45, 46], were significantly higher in the high absolute neutrophil group. Weisner et al. demonstrated that impaired CD4 Helper responses in immunosuppressed mice were responsible for switching between an eosinophilic and neutrophilic response to cryptococcal infection. A neutrophil response was associated with worse outcomes compared to eosinophil response in mice cryptococcal pulmonary infections.[47] Neutrophils activation as evidenced by high neutrophil CD64 levels has been found to play a role in systemic inflammation in HIV infected patients initiating ART[48] and may play a role in IRIS as they have been found to be activated in TB-IRIS. [49] Vlasova-St. Louis et al using genome wide transcriptomic analysis have found higher transcripts encoding markers for activated granulocytes, tissue infiltration and destruction among subjects that developed cryptococcal immune reconstitution syndrome.[50] Higher neutrophil percentages and neutrophil to lymphocyte ratios have also been found to be associated with mortality in patients with community acquired pneumonia and other chronic medical conditions.[51, 52] Curbelo et al hypothesize that the mortality is attributable to the sustained immune response rather than the infectious process.[51] Higher neutrophil count is associated with a Th1 response that is more robust and likely to cause more tissue damage.[10]

Prognostic markers are important in cryptococcal meningitis because cryptococcosis is highly prevalent and associated with high mortality, especially in sub-Saharan Africa.[53, 54] It is thought that these prognostic factors have regional variation [55–57]. A systematic review by Paquier et al has significantly highlighted these differences to include the economic status of the country, ART status, the induction therapies used, as well as the person’s HIV-status. The major prognostic factors in HIV-cryptococcal meningitis patients for long-term mortality were altered neurological status, low CD4 cell count, high CSF fungal/Cryptococcal antigen burden, older age at diagnosis and possibly immune reconstitution.[58] Lower CD4 counts <50 cells/mm^3^ in Uganda are associated with slightly higher mortality.[59] Despite the improvements in care, mortality is unchanged hence the need for new prognostic markers to identify those at risk and the causes of mortality.

While the higher neutrophil count is associated with mortality, the mechanism is not clear, and the pathophysiology is unknown. Higher neutrophils may be due to other undetected co-infections, but there was no statistical significance in the incidence of infections between the neutrophil groups in our study. Other conditions such as anemia and known mycobacterial infections which are known prognostic factors for death were adjusted for and not found to be significant [60]. A complete blood count is easily accessible and a routine test that is available even in many resource-limited settings. Getting a readily accessible test that may have implications for mortality in HIV-infected patients with cryptococcal meningitis is likely of value.

## Conclusion

Higher blood neutrophil counts in HIV-infected patients with cryptococcal meningitis were associated with mortality. Neutrophils role requires further investigation as to whether this a mediator directly contributing to mortality or merely a marker of underlying pathologies that increase mortality risk. Absolute neutropenia was not associated with mortality.

## Supporting information

S1 Checklist STROBE Checklist. (XLSX)

## References

[1] Boulware DR, Meya DB, Muzoora C, Rolfes MA, Huppler Hullsiek K, Musubire A, et al Timing of antiretroviral therapy after diagnosis of cryptococcal meningitis. The New England journal of medicine. 2014;370(26):2487–98. Epub 2014/06/26. 10.1056/NEJMoa1312884 ; PubMed Central PMCID: PMCPmc4127879. 24963568 PMC4127879

[2] Kambugu A, Meya DB, Rhein J, O'Brien M, Janoff EN, Ronald AR, et al Outcomes of cryptococcal meningitis in Uganda before and after the availability of highly active antiretroviral therapy. Clin Infect Dis. 2008;46(11):1694–701. Epub 2008/04/25. 10.1086/587667 18433339 PMC2593910

[3] Kwon-Chung KJ, Sorrell TC, Dromer F, Fung E, Levitz SM. Cryptococcosis: clinical and biological aspects. Medical mycology. 2000;38 Suppl 1:205–13. Epub 2001/02/24. . 11204147

[4] Davis JM, Huang M, Botts MR, Hull CM, Huttenlocher A. A Zebrafish Model of Cryptococcal Infection Reveals Roles for Macrophages, Endothelial Cells, and Neutrophils in the Establishment and Control of Sustained Fungemia. Infection and immunity. 2016;84(10):3047–62. Epub 2016/08/03. 10.1128/IAI.00506-16 27481252 PMC5038067

[5] Miller MF, Mitchell TG. Killing of *Cryptococcus neoformans* strains by human neutrophils and monocytes. Infection and immunity. 1991;59(1):24–8. Epub 1991/01/01. 1987038 10.1128/iai.59.1.24-28.1991 PMC257700

[6] Feldmesser M, Kress Y, Novikoff P, Casadevall A. Cryptococcus neoformans is a facultative intracellular pathogen in murine pulmonary infection. Infection and immunity. 2000;68(7):4225–37. Epub 2000/06/17. 10858240 10.1128/iai.68.7.4225-4237.2000 PMC101732

[7] Dong ZM, Murphy JW. Effects of the two varieties of Cryptococcus neoformans cells and culture filtrate antigens on neutrophil locomotion. Infection and immunity. 1995;63(7):2632–44. Epub 1995/07/01. 7790079 10.1128/iai.63.7.2632-2644.1995 PMC173353

[8] Ellerbroek PM, Lefeber DJ, van Veghel R, Scharringa J, Brouwer E, Gerwig GJ, et al O-acetylation of cryptococcal capsular glucuronoxylomannan is essential for interference with neutrophil migration. Journal of immunology (Baltimore, Md: 1950). 2004;173(12):7513–20. Epub 2004/12/09. . 15585878 10.4049/jimmunol.173.12.7513

[9] Richardson MD, White LJ, McKay IC, Shankland GS. Differential binding of acapsulate and encapsulated strains of Cryptococcus neoformans to human neutrophils. Journal of medical and veterinary mycology: bi-monthly publication of the International Society for Human and Animal Mycology. 1993;31(3):189–99. Epub 1993/01/01. . 8360810

[10] Mednick AJ, Feldmesser M, Rivera J, Casadevall A. Neutropenia alters lung cytokine production in mice and reduces their susceptibility to pulmonary cryptococcosis. European journal of immunology. 2003;33(6):1744–53. Epub 2003/06/05. 10.1002/eji.200323626 . 12778493

[11] Zhang M, Sun D, Shi M. Dancing cheek to cheek: Cryptococcus neoformans and phagocytes. SpringerPlus. 2015;4:410 Epub 2015/08/13. 10.1186/s40064-015-1192-3 26266081 PMC4531118

[12] Wozniak KL, Kolls JK, Wormley FL Jr. Depletion of neutrophils in a protective model of pulmonary cryptococcosis results in increased IL-17A production by gammadelta T cells. BMC immunology. 2012;13:65 Epub 2012/12/12. 10.1186/1471-2172-13-65 23216912 PMC3538069

[13] Guillot L, Carroll SF, Homer R, Qureshi ST. Enhanced innate immune responsiveness to pulmonary Cryptococcus neoformans infection is associated with resistance to progressive infection. Infection and immunity. 2008;76(10):4745–56. Epub 2008/08/06. 10.1128/IAI.00341-08 18678664 PMC2546841

[14] Zhang M, Sun D, Liu G, Wu H, Zhou H, Shi M. Real-time in vivo imaging reveals the ability of neutrophils to remove Cryptococcus neoformans directly from the brain vasculature. Journal of leukocyte biology. 2016;99(3):467–73. Epub 2015/10/03. 10.1189/jlb.4AB0715-281R . 26428677 PMC6608047

[15] Sun D, Zhang M, Liu G, Wu H, Zhu X, Zhou H, et al Real-Time Imaging of Interactions of Neutrophils with Cryptococcus neoformans Demonstrates a Crucial Role of Complement C5a-C5aR Signaling. Infection and immunity. 2016;84(1):216–29. Epub 2015/10/28. 10.1128/IAI.01197-15 26502909 PMC4693990

[16] Chiller T, Farrokhshad K, Brummer E, Stevens DA. Effect of granulocyte colony-stimulating factor and granulocyte-macrophage colony-stimulating factor on polymorphonuclear neutrophils, monocytes or monocyte-derived macrophages combined with voriconazole against Cryptococcus neoformans. Medical mycology. 2002;40(1):21–6. Epub 2002/02/28. . 11860010 10.1080/mmy.40.1.21.26

[17] Graybill JR, Bocanegra R, Lambros C, Luther MF. Granulocyte colony stimulating factor therapy of experimental cryptococcal meningitis. Journal of medical and veterinary mycology: bi-monthly publication of the International Society for Human and Animal Mycology. 1997;35(4):243–7. Epub 1997/07/01. . 9292420 10.1080/02681219780001221

[18] Coffey MJ, Phare SM, George S, Peters-Golden M, Kazanjian PH. Granulocyte colony-stimulating factor administration to HIV-infected subjects augments reduced leukotriene synthesis and anticryptococcal activity in neutrophils. The Journal of clinical investigation. 1998;102(4):663–70. Epub 1998/08/26. 10.1172/JCI2117 9710433 PMC508927

[19] Vecchiarelli A, Monari C, Baldelli F, Pietrella D, Retini C, Tascini C, et al Beneficial effect of recombinant human granulocyte colony-stimulating factor on fungicidal activity of polymorphonuclear leukocytes from patients with AIDS. J Infect Dis. 1995;171(6):1448–54. Epub 1995/06/01. . 7539473 10.1093/infdis/171.6.1448

[20] Aratani Y, Kura F, Watanabe H, Akagawa H, Takano Y, Ishida-Okawara A, et al Contribution of the myeloperoxidase-dependent oxidative system to host defence against Cryptococcus neoformans. Journal of medical microbiology. 2006;55(Pt 9):1291–9. Epub 2006/08/18. 10.1099/jmm.0.46620-0 . 16914663

[21] Rhein J, Morawski BM, Hullsiek KH, Nabeta HW, Kiggundu R, Tugume L, et al Efficacy of adjunctive sertraline for the treatment of HIV-associated cryptococcal meningitis: an open-label dose-ranging study. The Lancet Infectious diseases. 2016;16(7):809–18. Epub 2016/03/14. 10.1016/S1473-3099(16)00074-8 ; PubMed Central PMCID: PMC4927382. 26971081 PMC4927382

[22] Manga G, Calin GA, Manuc M, Droc G, Tudor S. New Definitions of Sepsis and the Quest for Specific Biomarkers. Are the miRNAs the Answer? Chirurgia (Bucharest, Romania: 1990). 2018;113(4):464–8. Epub 2018/09/06. 10.21614/chirurgia.113.4.464 . 30183576

[23] Bahr NC, Nuwagira E, Evans EE, Cresswell FV, Bystrom PV, Byamukama A, et al Diagnostic accuracy of Xpert MTB/RIF Ultra for tuberculous meningitis in HIV-infected adults: a prospective cohort study. The Lancet Infectious diseases. 2018;18(1):68–75. Epub 2017/09/19. 10.1016/S1473-3099(17)30474-7 ; PubMed Central PMCID: PMC5739874. 28919338 PMC5739874

[24] Bahr NC, Tugume L, Rajasingham R, Kiggundu R, Williams DA, Morawski B, et al Improved diagnostic sensitivity for tuberculous meningitis with Xpert((R)) MTB/RIF of centrifuged CSF. The international journal of tuberculosis and lung disease: the official journal of the International Union against Tuberculosis and Lung Disease. 2015;19(10):1209–15. Epub 2015/10/16. 10.5588/ijtld.15.0253 ; PubMed Central PMCID: PMC4768484. 26459535 PMC4768484

[25] Zeh C, Amornkul PN, Inzaule S, Ondoa P, Oyaro B, Mwaengo DM, et al Population-based biochemistry, immunologic and hematological reference values for adolescents and young adults in a rural population in Western Kenya. PloS one. 2011;6(6):e21040 Epub 2011/06/30. 10.1371/journal.pone.0021040 21713038 PMC3119664

[26] Eller LA, Eller MA, Ouma B, Kataaha P, Kyabaggu D, Tumusiime R, et al Reference intervals in healthy adult Ugandan blood donors and their impact on conducting international vaccine trials. PloS one. 2008;3(12):e3919 Epub 2008/12/17. 10.1371/journal.pone.0003919 19079547 PMC2593783

[27] Kibaya RS, Bautista CT, Sawe FK, Shaffer DN, Sateren WB, Scott PT, et al Reference ranges for the clinical laboratory derived from a rural population in Kericho, Kenya. PloS one. 2008;3(10):e3327 Epub 2008/10/04. 10.1371/journal.pone.0003327 18833329 PMC2553265

[28] Chisale MR, Kumwenda P, Ngwira M, M'Baya B, Chosamata BI, Mwapasa V. A pilot study to determine the normal haematological indices for young Malawian adults in Blantyre, Malawi. Malawi medical journal: the journal of Medical Association of Malawi. 2015;27(3):96–100. Epub 2015/12/31. 26715954 10.4314/mmj.v27i3.5 PMC4688870

[29] Erhabor O, Ejele OA, Nwauche CA, Buseri FI. Some haematological parameters in human immunodeficiency virus (HIV) infected Africans: the Nigerian perspective. Nigerian journal of medicine: journal of the National Association of Resident Doctors of Nigeria. 2005;14(1):33–8. Epub 2005/04/19. . 15832640 10.4314/njm.v14i1.37132

[30] Levine AM, Karim R, Mack W, Gravink DJ, Anastos K, Young M, et al Neutropenia in human immunodeficiency virus infection: data from the women's interagency HIV study. Archives of internal medicine. 2006;166(4):405–10. Epub 2006/03/01. 10.1001/archinte.166.4.405 . 16505259

[31] Firnhaber C, Smeaton L, Saukila N, Flanigan T, Gangakhedkar R, Kumwenda J, et al Comparisons of anemia, thrombocytopenia, and neutropenia at initiation of HIV antiretroviral therapy in Africa, Asia, and the Americas. International journal of infectious diseases: IJID: official publication of the International Society for Infectious Diseases. 2010;14(12):e1088–92. Epub 2010/10/22. 10.1016/j.ijid.2010.08.002 ; PubMed Central PMCID: PMCPmc3021118. 20961784 PMC3021118

[32] Mora DJ, Fortunato LR, Andrade-Silva LE, Ferreira-Paim K, Rocha IH, Vasconcelos RR, et al Cytokine profiles at admission can be related to outcome in AIDS patients with cryptococcal meningitis. PloS one. 2015;10(3):e0120297 Epub 2015/03/24. 10.1371/journal.pone.0120297 25799044 PMC4370646

[33] Rolfes MA, Hullsiek KH, Rhein J, Nabeta HW, Taseera K, Schutz C, et al The effect of therapeutic lumbar punctures on acute mortality from cryptococcal meningitis. Clin Infect Dis. 2014;59(11):1607–14. Epub 2014/07/25. 10.1093/cid/ciu596 ; PubMed Central PMCID: PMC4441057. 25057102 PMC4441057

[34] Jarvis JN, Bicanic T, Loyse A, Namarika D, Jackson A, Nussbaum JC, et al Determinants of mortality in a combined cohort of 501 patients with HIV-associated Cryptococcal meningitis: implications for improving outcomes. Clin Infect Dis. 2014;58(5):736–45. Epub 2013/12/10. 10.1093/cid/cit794 ; PubMed Central PMCID: PMC3922213. 24319084 PMC3922213

[35] Leopold Wager CM, Hole CR, Wozniak KL, Wormley FL Jr. Cryptococcus and Phagocytes: Complex Interactions that Influence Disease Outcome. Frontiers in microbiology. 2016;7:105 Epub 2016/02/24. 10.3389/fmicb.2016.00105 26903984 PMC4746234

[36] Heung LJ. Innate Immune Responses to Cryptococcus. Journal of fungi (Basel, Switzerland). 2017;3(3). Epub 2017/09/25. 10.3390/jof3030035 28936464 PMC5604851

[37] Feretzaki M, Hardison SE, Wormley FL Jr., Heitman J. Cryptococcus neoformans hyperfilamentous strain is hypervirulent in a murine model of cryptococcal meningoencephalitis. PloS one. 2014;9(8):e104432 Epub 2014/08/06. 10.1371/journal.pone.0104432 25093333 PMC4122496

[38] Leopold Wager CM, Hole CR, Wozniak KL, Olszewski MA, Wormley FL Jr. STAT1 signaling is essential for protection against Cryptococcus neoformans infection in mice. Journal of immunology (Baltimore, Md: 1950). 2014;193(8):4060–71. Epub 2014/09/10. 10.4049/jimmunol.1400318 25200956 PMC4185263

[39] Shih CC, Chen YC, Chang SC, Luh KT, Hsieh WC. Cryptococcal meningitis in non-HIV-infected patients. QJM: monthly journal of the Association of Physicians. 2000;93(4):245–51. Epub 2000/04/29. . 10787453 10.1093/qjmed/93.4.245

[40] Scriven JE, Graham LM, Schutz C, Scriba TJ, Wilkinson KA, Wilkinson RJ, et al A Glucuronoxylomannan-Associated Immune Signature, Characterized by Monocyte Deactivation and an Increased Interleukin 10 Level, Is a Predictor of Death in Cryptococcal Meningitis. J Infect Dis. 2016;213(11):1725–34. Epub 2016/01/16. 10.1093/infdis/jiw007 ; PubMed Central PMCID: PMC4857465. 26768248 PMC4857465

[41] Bisson GP, Ramchandani R, Miyahara S, Mngqibisa R, Matoga M, Ngongondo M, et al Risk factors for early mortality on antiretroviral therapy in advanced HIV-infected adults. AIDS (London, England). 2017;31(16):2217–25. Epub 2017/07/26. 10.1097/qad.0000000000001606 28742529 PMC5633516

[42] Rajasingham R, Williams D, Meya DB, Meintjes G, Boulware DR, Scriven J. Nosocomial drug-resistant bacteremia in 2 cohorts with cryptococcal meningitis, Africa. Emerging infectious diseases. 2014;20(4):722–4. Epub 2014/03/25. 10.3201/eid2004.131277 ; PubMed Central PMCID: PMCPmc3966372. 24655747 PMC3966372

[43] Kerkhoff AD, Wood R, Lowe DM, Vogt M, Lawn SD. Blood neutrophil counts in HIV-infected patients with pulmonary tuberculosis: association with sputum mycobacterial load. PloS one. 2013;8(7):e67956 Epub 2013/07/23. 10.1371/journal.pone.0067956 23874476 PMC3706476

[44] Lowe DM, Bandara AK, Packe GE, Barker RD, Wilkinson RJ, Griffiths CJ, et al Neutrophilia independently predicts death in tuberculosis. The European respiratory journal. 2013;42(6):1752–7. Epub 2013/10/12. 10.1183/09031936.00140913 24114967 PMC4176760

[45] Yan S, Chen L, Wu W, Li Z, Fu Z, Zhang H, et al Paradoxical immune reconstitution inflammatory syndrome associated with cryptococcal meningitis in China: a 5-year retrospective cohort study. Clinical microbiology and infection: the official publication of the European Society of Clinical Microbiology and Infectious Diseases. 2015;21(4):379.e11–4. Epub 2015/02/07. 10.1016/j.cmi.2014.11.011 . 25658526

[46] Boulware DR, Meya DB, Bergemann TL, Wiesner DL, Rhein J, Musubire A, et al Clinical features and serum biomarkers in HIV immune reconstitution inflammatory syndrome after cryptococcal meningitis: a prospective cohort study. PLoS medicine. 2010;7(12):e1000384 Epub 2011/01/22. 10.1371/journal.pmed.1000384 21253011 PMC3014618

[47] Wiesner DL, Smith KD, Kashem SW, Bohjanen PR, Nielsen K. Different Lymphocyte Populations Direct Dichotomous Eosinophil or Neutrophil Responses to Pulmonary Cryptococcus Infection. Journal of immunology (Baltimore, Md: 1950). 2017;198(4):1627–37. Epub 2017/01/11. 10.4049/jimmunol.1600821 28069805 PMC5296217

[48] Mitsumoto-Kaseida F, Murata M, Ura K, Takayama K, Hiramine S, Shimizu M, et al The Expression Level of Neutrophil CD64 Is a Useful Marker of Systemic Inflammation Associated with HIV Infection. AIDS research and human retroviruses. 2017;33(2):147–56. Epub 2016/10/21. 10.1089/AID.2016.0107 . 27762593

[49] Nakiwala JK, Walker NF, Diedrich CR, Worodria W, Meintjes G, Wilkinson RJ, et al Neutrophil activation and enhanced release of granule products in HIV-TB immune reconstitution inflammatory syndrome. Journal of acquired immune deficiency syndromes (1999). 2017 Epub 2017/11/15. 10.1097/qai.0000000000001582 . 29135655 PMC5765966

[50] Vlasova-St Louis I, Chang CC, Shahid S, French MA, Bohjanen PR. Transcriptomic Predictors of Paradoxical Cryptococcosis-Associated Immune Reconstitution Inflammatory Syndrome. Open forum infectious diseases. 2018;5(7):ofy157 Epub 2018/07/25. 10.1093/ofid/ofy157 30038928 PMC6051466

[51] Curbelo J, Luquero Bueno S, Galvan-Roman JM, Ortega-Gomez M, Rajas O, Fernandez-Jimenez G, et al Inflammation biomarkers in blood as mortality predictors in community-acquired pneumonia admitted patients: Importance of comparison with neutrophil count percentage or neutrophil-lymphocyte ratio. PloS one. 2017;12(3):e0173947 Epub 2017/03/17. 10.1371/journal.pone.0173947 28301543 PMC5354424

[52] Isaac V, Wu CY, Huang CT, Baune BT, Tseng CL, McLachlan CS. Elevated neutrophil to lymphocyte ratio predicts mortality in medical inpatients with multiple chronic conditions. Medicine. 2016;95(23):e3832 Epub 2016/06/10. 10.1097/MD.0000000000003832 27281085 PMC4907663

[53] Rajasingham R, Smith RM, Park BJ, Jarvis JN, Govender NP, Chiller TM, et al Global burden of disease of HIV-associated cryptococcal meningitis: an updated analysis. The Lancet Infectious diseases. 2017;17(8):873–81. Epub 2017/05/10. 10.1016/S1473-3099(17)30243-8 28483415 PMC5818156

[54] Antinori S. New Insights into HIV/AIDS-Associated Cryptococcosis. Isrn aids. 2013;2013:471363 Epub 2013/09/21. 10.1155/2013/471363 24052889 PMC3767198

[55] Moosa MY, Coovadia YM. Cryptococcal meningitis in Durban, South Africa: a comparison of clinical features, laboratory findings, and outcome for human immunodeficiency virus (HIV)-positive and HIV-negative patients. Clin Infect Dis. 1997;24(2):131–4. Epub 1997/02/01. . 9114135 10.1093/clinids/24.2.131

[56] Sloan DJ, Parris V. Cryptococcal meningitis: epidemiology and therapeutic options. Clinical epidemiology. 2014;6:169–82. Epub 2014/05/30. 10.2147/CLEP.S38850 24872723 PMC4026566

[57] Majumder S, Mandal SK, Bandyopadhyay D. Prognostic markers in AIDS-related cryptococcal meningitis. The Journal of the Association of Physicians of India. 2011;59:152–4. Epub 2011/07/15. . 21751623

[58] Pasquier E, Kunda J, De Beaudrap P, Loyse A, Temfack E, Molloy SF, et al Long-term Mortality and Disability in Cryptococcal Meningitis: A Systematic Literature Review. Clin Infect Dis. 2018;66(7):1122–32. Epub 2017/10/14. 10.1093/cid/cix870 . 29028957

[59] Tugume L, Rhein J, Hullsiek KH, Mpoza E, Kiggundu R, Ssebambulidde K, et al HIV-associated Cryptococcal Meningitis Occurring at Relatively Higher CD4 counts. J Infect Dis. 2018:*In Press* Epub 2018/10/17. 10.1093/infdis/jiy602 . 30325463 PMC6387427

[60] Wenger JD, Whalen CC, Lederman MM, Spech TJ, Carey JT, Tomford JW, et al Prognostic factors in acquired immunodeficiency syndrome. Journal of general internal medicine. 1988;3(5):464–70. . 3262733 10.1007/BF02595923

